# Orthodontic tooth movement of total buccally blocked-out canine: a case report

**DOI:** 10.4076/1757-1626-2-7245

**Published:** 2009-07-30

**Authors:** Hessa M Alkhal, Bakr Rabie, Ricky W K Wong

**Affiliations:** 1 Dental Department, Doha-Qatar Hamad Medical CorporationDohaQatar; 2 Orthodontics, Faculty of Dentistry, 2/F Prince Philip Dental Hospital34 Hospital Road, Hong Kong SARChina

## Abstract

Orthodontic tooth movement of total buccally blocked-out canine is usually difficult as it is related with the problems of severe crowding, midline deviation, involvement of long root movement and risk of gingival recession. A case report was presented to illustrate the treatment principles. It demonstrated with careful planning in extraction sequence and orthodontic mechanics to deliver light, controlled force, condition of totally blocked out canine could be corrected with good results.

## Introduction

Orthodontic management of a total buccally blocked-out canine is very challenging as it is related to a variety of problems. These problems and the strategies to overcome them were listed below.

First, there is usually severe crowding, at least in the canine region. To overcome this, space is needed to be created for alignment. Extractions are usually needed. The anchorage situation is usually severe. This problem may be controlled by anchorage reinforcement measures such as a palatal arch or a Nance button.

Second, in some cases one side of the crowding (the side of the blocked out canine) may be more severe than the other side, in these cases the dental midline is usually shifted to the crowded side. This midline correction can be facilitated by delaying extraction on the crowded side during orthodontic midline correction. This also reduces the chance of the crowded canine to drop in place more to the crowded side than it should be which makes subsequent midline correction and to obtain a decent occlusion difficult.

Third, the condition usually requires substantial amount of bodily movement of canine which is difficult to perform because the canine has a long and bulbous root. This morphology makes bodily movement of the canine time-consuming, difficult to control and often results in root resorption. Even when orthodontic forces are applied in a desired direction, it is difficult to produce the amount of root movement required because a large hyalinised layer will be created [1]. In addition, the canine root is usually close to the cortical bone of the maxilla, an area of reduced vascularisation. This results in delayed bone remodeling and tooth movement. In order to produce efficient canine root movement, very light orthodontic force will be needed. This can be achieved by sectional wire with frictionless mechanic coupled with slight activation during canine retraction stage, and using long span of wire (with increased interbracket width and increased flexibility) by differential bonding of the teeth during alignment stage.

Fourth, the buccal bone covering the buccally placed canine root is usually thin. Therefore, palatal root torque is needed for the canine to increase the buccal bone thickness, decrease the risk of bone dehiscence and decrease the risk of gingival recession [2]. In addition, the canine should be allowed to erupt in place naturally rather than to extrude it as this may lead to gingival recession [2].

To illustrate the above points, an orthodontic management of a totally buccally blocked-out canine case is reported.

## Case presentation

A 14 years old Chinese female came for orthodontic treatment. On diagnosis, she had a problem list as follows:

**Extra-oral condition:** Convex profile, acute nasolabial angle, increased lower facial height, increased mandibular plane angle, retrusive mandible and short ramus.

**Figure 1.**
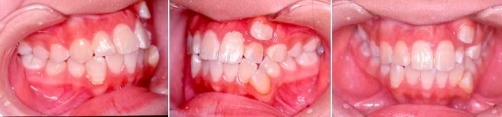
Totally blocked out 23. Palatal displaced 22. Buccally displaced 33.

**Intra-oral condition** (Figure 1)

**Sagittal:** Molar Class III on right side and canines are unclassified on left side, decrease overjet. Crossbite tooth left maxillary lateral incisor (22) against left mandibular lateral incisor (32).

**Vertical:** Increased lower facial height and decreased overbite.

**Transversal:** Lower and upper center lines are shifted to left side (4 mm and 3 mm respectively), upper 8.5 mm crowding, lower 9 mm crowding, buccal displacement of left maxillary canine (23) (totally blocked out) and left mandibular canine (33), palatal displacement of left maxillary lateral incisor (22).

**The treatment plan was:**

- Oral hygiene instruction
- Full mouth scaling and prophylaxis
- Fixed orthodontic appliance with extractions of teeth right maxillary first premolar (14), left maxillary first premolar (24), left mandibular first premolar (34), right mandibular first premolar (44) with maximum anchorage
- Fixed lingual retainer right maxillary canine (13) - left maxillary canine (23) and left mandibular canine (33) - right mandibular canine (43) with removable wraparound retainers
- Review third molars eruption

**Figure 2.**
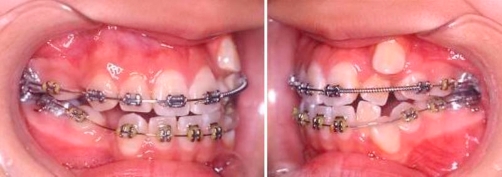
Upper arch: 0.017 inch × 0.025 inch stainless steel archwire, open coil to create space for 22, 23 and to correct midline. Lower arch: 0.014 inch NiTi archwire.

**Treatment performed** Orthodontic treatment was started with initial alignment of teeth with upper and lower 0.014 inch NiTi archwires, left maxillary first premolar (14) and right mandibular first premolar (44) were extracted. Three months into treatment, the strategy was to create space for left maxillary lateral incisor (22) and left maxillary canine (23) and to correct the upper midline. A 0.017 inch × 0.025 inch stainless steel archwire was placed on the upper arch, with an open coil spring to create space for left maxillary lateral incisor (22), left maxillary canine (23) and to correct midline. Lower arch: 0.014 inch NiTi archwire to continue alignment (Figure 2).

**Figure 3.**
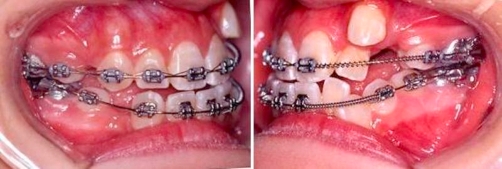
Upper arch: Extraction of 24. 23 allowed to drift down. Lower arch: 0.017 inch × 0.025 inch stainless steel archwire. Open coil to create space for 32, 33 and to correct lower midline.

Five months into treatment, in the upper arch, extraction of left maxillary first premolar (24) was performed to allow left maxillary canine (23) to erupt naturally. In the lower arch, a 0.017 inch × 0.025 inch stainless steel archwire was placed. An open coil spring was inserted to create space for left mandibular lateral incisor (32) and left mandibular canine (33) and to correct lower midline, similar to the upper arch (Figure 3).

**Figure 4.**
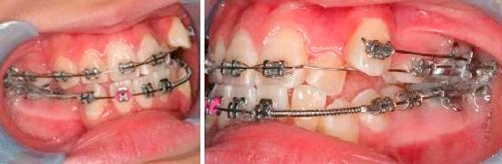
Upper arch: 0.018 inch stainless steel main archwire. 0.017 inch × 0.025 inch TMA sectional wire with closing loop for distalisation, palatal root torquing and then extrusion of 23.

Eight months into treatment, in the upper arch, the strategy was to distalise the canine with light force and increase the nearby buccal bone thickness. While maintaining the upper arch with a 0.018 inch stainless steel archwire; a 0.017 inch × 0.025 inch TMA sectional wire with closing loop for was used for distalisation, palatal root torquing and then extrusion of left maxillary canine (23) (Figure 4).

**Figure 5.**
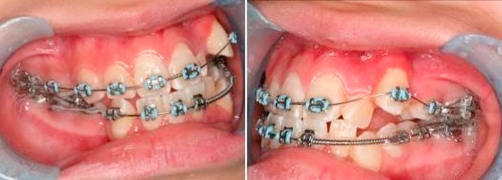
Upper arch: 0.014 inch thermal NiTi archwire to align 23. 22 not bonded. Lower arch: 0.019 inch × 0.025 inch stainless steel archwire. Open coil to create space for 32, 33 and to correct lower midline. Power chain to retract 43.

Ten months into treatment, in the upper arch, a 0.014 inch thermal NiTi archwire was used to align (23). Left maxillary lateral incisor (22) was not included for alignment at this stage to increase the interbracket distance between (21) and left maxillary canine (23) and to increase the flexibility of the wire. In the lower arch, a 0.019 inch × 0.025 inch stainless steel archwire was inserted, an open coil spring was used to create space for left mandibular lateral incisor (32), left mandibular canine (33) and to correct lower midline. On the right side, a power chain was used to retract right mandibular canine (43) (Figure 5).

**Figure 6.**
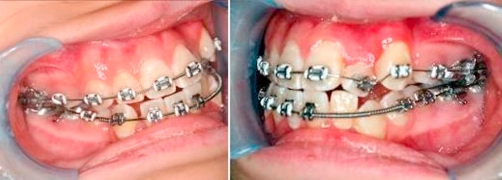
22 included for alignment.

Twelve months into treatment, left maxillary lateral incisor (22) was included for alignment (Figure 6).

**Figure 7.**
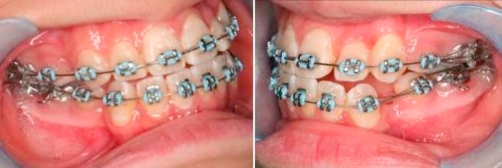
Extraction of 34. 0.014 inch NiTi archwire to align 32, 33.

Thirteen months into treatment, extraction of left mandibular first premolar (34) was performed, this delay in extraction allowed more efficient correction of the midline. A 0.014 inch NiTi archwire was used in the lower arch to align left mandibular lateral incisor (32), left mandibular canine (33) (Figure 7).

**Figure 8.**
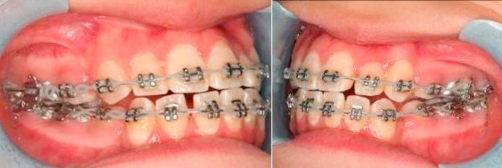
Lower arch: 0.016 inch stainless steel archwire. Close spaces.

Fifteen months into treatment, in the lower arch a 0.016 inch stainless steel archwire was used for further alignment and to start closing spaces (Figure 8).

**Figure 9.**
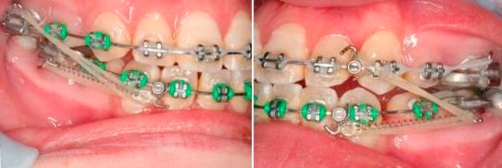
0.019 inch × 0.025 inch stainless steel archwire for arch coordination and space closure.

Eighteen months into treatment, upper and lower 0.019 inch × 0.025 inch stainless steel archwires were used for arch coordination and space closure (Figure 9).

**Figure 10.**
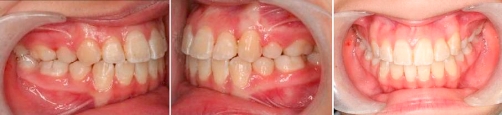
Finished occlusion.

Twenty-seven months into treatment, the case was debonded, the teeth were in well-interdigitated occlusion (Figure 10).

## Conclusion

This case report has demonstrated with careful planning in extraction sequence and orthodontic mechanics to deliver light, controlled force, condition of totally blocked out canine can be corrected with good results.

## Abbreviations

NiTi: Nickel Titanium
TMA: Titanium Molybdenum Alloy
